# Study on the effect of koumiss on reactivation of *Toxoplasma gondii* infection

**DOI:** 10.3389/fnut.2022.1032271

**Published:** 2022-10-20

**Authors:** Xinlei Yan, Yufei Sun, Xiuli Yu, Jialu Gao, Hejing Wang, Ru Liang, Wenying Han, Xindong Jin, Wenhui Guo, Pufang Liu, Jia Chen

**Affiliations:** ^1^College of Food Science and Engineering, Inner Mongolia Agricultural University, Hohhot, China; ^2^College of Food Science and Engineering, Northwest Agriculture and Forestry University, Xianyang, Shaanxi, China; ^3^Department of Pediatrics, Inner Mongolia Maternal, Child Health Hospital, Hohhot, China

**Keywords:** koumiss, reactivation of *Toxoplasma gondii* infection, immune factor, intestinal microbiota, *Bifidobacterium*

## Abstract

*Toxoplasma gondii* is an obligate intracellular parasite that infects nucleated cells of all warm-blooded animals, and most patients have latent infections. The latent infection will be reactivated in the immunocompromised or immunocompromised individuals, which will lead to severe toxoplasmosis. At present, less research has been focused on the reactivation of *T. gondii* infection. Koumiss is a kind of fermented milk made from fresh mare’s milk through natural fermentation that can be applied to clinical and rehabilitation medicine to mitigate the development of various diseases due to its unique functional characteristics. In this study, we explored the antagonistic effect of koumiss on reactivation of *T. gondii* infection. Mice were treated with dexamethasone to establish a reactivation model after infection with *T. gondii* and then treated with koumiss. The survival rate, SHIRPA test, serum cytokine levels, organ parasite burden and intestinal microbiota were measured, respectively. Our results showed that koumiss treatment improved the clinical symptoms of mice, significantly reduced the organ parasite burden of mice, and improved the composition and structure of intestinal flora. This study provides new evidence for the alleviation and treatment of toxoplasmosis and provides a novel idea for the development and utilization of koumiss.

## Introduction

*Toxoplasma gondii* (*T. gondii*) is an obligate intracellular parasite that is widespread worldwide and can cause encephalitis, enteritis, and pregnancy loss. Nearly all warm-blooded creatures are at risk of infection, including humans (1, 2). *T. gondii* has a very complex life cycle because the reproduction and development of the parasite require intermediate hosts and definitive hosts (3). Infection may occur through consumption of food or water contaminated with cat feces carrying infected oocysts, raw meat products that have formed *T. gondii* tissue cysts, or undercooked raw meat. Maternal infection with *T. gondii* during pregnancy can cause congenital infections such as teratogenicity and stillbirth (4). According to previous reports, approximately 30% of the world’s population is infected with *T. gondii* and suffers from toxoplasmosis, and most patients have latent infection (5).

*T. gondii* is a class of opportunistic pathogenic parasitic protozoa, and the damage caused to the host by infection with *T. gondii* is closely related to the state of the host’s immune system. When *T. gondii* infects individuals with normal immunity, the clinical manifestations are mostly stable latent long-term infection. In immunocompromised or immunodeficient individuals, such as patients with tumors or AIDS, latent infection will be activated. *T. gondii* tachyzoites can multiply rapidly and cause disseminated lesions in the body, even resulting in death (6). *T. gondii* coinfection occurs in 5–10% of patients with AIDS, and the resulting encephalopathy is one of the causes of death in patients with AIDS (7). Previous studies have shown that *T. gondii* infection can cause neurological diseases (8), and many studies have reported a higher prevalence of *T. gondii* antibodies in schizophrenia patients than in the normal population, which indicated that *T. gondii* infection can seriously threaten human health (9). However, less research has been focused on the reactivation of *T. gondii* infection when the host has a low immune system function after the host is infected with *T. gondii*. To date, there is no specific vaccine or drug for treatment of *T. gondii* infection, clinical treatment of toxoplasmosis usually involves the use of pyrimethamine or sulfonamides, but there is a risk of adverse effects such as diarrhea, nausea, vomiting, and even the risk of teratogenicity with etanercept. Reactivated toxoplasmosis introduces combination antiretroviral therapy (cART), which reduces the incidence but still has the potential for reactivation of toxoplasmosis (10). Therefore, there is an urgent need to find a safe and effective method to alleviate *T. gondii* infection.

The composition and function of the gut microbiota are closely related to the development of disease after human infection with parasites (11). The changes in the composition and species of the microbiota may prevent the infection process of intestinal parasites. Recently, the bidirectional link between the gut microbiota and the central nervous system has received much attention, and the gut microbiota has been implicated in the pathological mechanisms of many behaviors and psychiatric disorders through the “gut-brain axis” (12). Mice infected with *Trichuris muris* exhibit anxiety-like behavior but can be restored to normal by treatment with the probiotic strain *Bifidobacterium longum* (13). However, the effect of changes in gut microbiota on reactivation of *T. gondii* in mice is unclear.

Koumiss is a kind of traditional acid-fermented milk with a unique aroma and low ethanol content that is made of fresh mare milk through naturally fermented by microorganisms such as lactic acid bacteria and yeasts (14). It is a very popular dairy food for the people of Mongolia, Kazakhstan, Kyrgyzstan, and some regions of Russia and Bulgaria (15). Many studies have reported that it may affect and alleviate the development of various diseases, such as chronic gastritis and cardiovascular system diseases, by changing the composition of gut microbiota due to its rich microbial resources and nutrients (16, 17). The nomadic people in Inner Mongolia use koumiss as a traditional Mongolian medicine to treat and heal intestinal dyspepsia, hypertension, tuberculosis and dyslipidemia (18–20).

Dexamethasone (DEX) is a glucocorticoid that is widely used clinically to treat inflammatory and autoimmune diseases. However, long-term use of DEX can make patients more susceptible to *T. gondii* infection, resulting in acute toxoplasmosis (21). In this study, *T. gondii* was used to cause chronic infection in mice. After a stable latent infection was formed, the mice were treated with DEX to establish a reactivation of *T. gondii* infection model. Koumiss was used to treat the mice in order to explore the effect of koumiss on reactivation of *T. gondii* infection in mice. Our data showed an alleviating effect of koumiss on reactivation of *T. gondii* infection, which provides a novel idea and theoretical basis for the treatment method of anti-*T. gondii* infection.

## Materials and methods

### Animals and parasites

Forty-eight 6-week-old female BALB/c mice with a body weight of 15 to 18 g without specific pathogens were purchased from SPF (Beijing) Biotechnology Co., Ltd., Beijing, China. All mice were given free access to food and water. Sterile rat chow, drinking water, and bedding were changed daily, and the mice were housed under a 12 h light/dark cycle. The animal experiment protocol was approved by the Laboratory Animal Welfare and Animal Experiment Ethics Inspection Committee of Inner Mongolia Agricultural University (approval No.: NND2021069). The *T. gondii* PRU strain was donated by the National Animal Protozoa Laboratory of China Agricultural University, passaged and preserved serially in Kunming mice. Koumiss samples and fresh mare’s milk samples obtained from the Abaga Banner of Xilin Gol League were used in their original form (fermentation temperature: 18–20°C, fermentation time: 72 h, pH value: 3.88). After a 1-week acclimation period, mice were randomly divided into the Control group (C, *n* = 12), Infection group (G, *n* = 12), Koumiss group (S, *n* = 12), and Mare milk group (X, *n* = 12). Then, the G, S, and X groups were infected orally with 3 *T. gondii* cysts in 200 μL PBS, while the C group was given the same volume of PBS alone. After 28 days, DEX (8 mg/L) was added to the drinking water of mice to establish the reactivation of *T. gondii* infection model in mice. The mice in Groups C, G, S and X were fed PBS, PBS, koumiss and fresh mare’s milk (5 mL/kg) daily, respectively.

### Sample collection

Nine mice were randomly selected from each group to calculate the survival rate after establishing the reactivated *T. gondii* infection model, the remaining three mice were conducted the experiment of body weights recorded, and SHIRPA determined at 7 dpi. The feces of the mice were collected aseptically and blood was collected from the eyeballs. The mice were then sacrificed by cervical dislocation and the organs were collected to measure the organ parasite burden. The remaining mice completed the survival test. The feces were frozen in liquid nitrogen and stored at −80°C until assayed.

### Evaluation of SHIRPA

The modified SHIRPA (22) procedure was used to describe and characterize the behavior and symptoms of mice after establishing the reactivation of *T. gondii* infection model. The scoring items are as follows: standing hair, abdominal peristalsis, weight loss, diarrhea, watery eyes (orbital wetness), squinting, slower movement speed, avoidance from touch, involuntary tremors, decreased grip strength, curled up, deepened and accelerated breathing 1 point is awarded for any one of the above, out of a total of 12 points.

### Determination of serum cytokine levels

Blood samples collected from mouse eyeballs were left at room temperature for 1 h, in a constant temperature incubator at 37°C for 1 h, and in a refrigerator at 4°C for 1 h, then centrifuged at 1,000 × *g* for 20 min, and the supernatant was aspirated and stored at −20°C for preservation. The levels of the serum cytokines IL-4, IL-10, IFN-γ, and TNF-α were measured using enzyme-linked immunosorbent assay (ELISA) kits (Production lot number: 03/2021, Tiangen, Beijing) as per the manufacturer’s instructions.

### Determination of organ parasite burden

The parasite burden of *T. gondii* in different organs of mice was determined by reverse transcription-polymerase chain reaction (RT-PCR), and genomic DNA was extracted using a tissue genomic DNA extraction kit as per the manufacturer’s instructions. For PCR amplification of the extracted genomic DNA, the *T. gondii* B1 gene was the target gene, the primer sequence was F (5′-TCCTTCGTCCGTCGTAAT-3′) and R (5′-TTCTTCAGCCGTCTTGTG-3′) (23), and the primer sequence of the internal control β-actin was F (5′-TTGCTGACAGGATGCAGAAG-3′) and R (5′-ACATCTGCTGGAAGGTGGAC-3′) (24). PCR was designed in a 25 μL reaction system that contained 12.5 μL 2 × Taq PCR Master Mix, 1 μL of each forward and reverse primer, 1 μL DNA template, and 9.5 μL ddH2O. The PCR conditions were as follows: initial denaturation at 94°C for 3 min, followed by 35 cycles of denaturation at 94°C for 30 s, annealing at 60°C for 30 s, and elongation at 72°C for 30 s, with a final elongation stage at 72°C for 5 min. The PCR products were detected by 1% agarose gel electrophoresis. Then, RT-PCR amplification was performed in a 20 μL reaction system that contained 10 μL SYBR Green Master Mix, 0.8 μL of each forward and reverse primer, 2 μL DNA template, and 6.4 μL ddH2O. The reaction conditions were the same as those for PCR amplification.

### Detection of intestinal microbiota

For fecal genomic DNA extraction and PCR amplification, the V3-V4 region of the 16S rRNA gene was sequenced by using the cetyltrimethylammonium ammonium bromide (CATB) method to determine the composition of the intestinal microbiota. Amplification was performed using primers with the following sequences: the forward primer was 515F (5′-GTGCCAGCMGCCGCGGTAA-3′) and the reverse primer was 806R (5′-GGACTACHVGGTWTCTAAT-3′). The PCR conditions were as follows: initial denaturation at 98°C for 1 min, followed by 30 cycles of denaturation at 98°C for 10 s, annealing at 50°C for 30 s, and elongation at 72°C for 30 s, with a final elongation stage at 72°C for 5 min. The PCR products were determined by 2% agarose gel electrophoresis and purified with a Qiagen Gel Extraction Kit (No. 28704, Qiagen, Germany).

### Library construction and sequencing

The library was constructed using the TruSeq^®^ DNA PCR-Free Sample Preparation Kit (Illumina, USA), and then sequenced on the Illumina NovaSeq sequencing platform to generate 250 bp paired-end reads, and the measurement was repeated three times for each sample. The obtained reads were spliced with FLASH^1^ and filtered with QIIME.^2^ Tags were compared with reference databases (Silva database),^3^ and the chimeric sequence was removed to obtain the effective tags.

### Bioinformatics analysis

The effective tags of all samples were clustered with 97% similarity as operational taxonomic units (OTUs) using the UPARSE algorithm.^4^ The minimum amount of data in the sample was used as the standard for homogenization α diversity analysis and β diversity analysis. The taxonomic information was obtained by species annotation analysis, and the community composition of the samples was counted individually at each classification level, including the Kingdom, Phylum, Class, Order, Family, Genus, and Species levels.

### Data statistics and analysis

GraphPad Prism software was used for data analyses and plots. Statistical analysis was performed using one-way analysis of variance (ANOVA) followed by Tukey’s test using SPSS 21.0 software (SPSS Inc., Chicago, IL, USA). All data are expressed as the means ± standard deviations (SD). *P* < 0.05 was considered to be statistically significant.

## Results

### Effects of koumiss on the survival rate of mice with reactivation of *Toxoplasma gondii* infection

**FIGURE 1:**
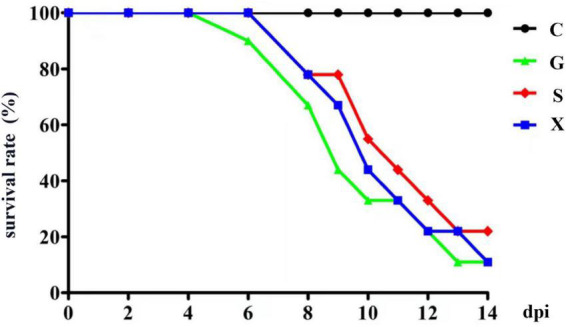
The survival rate of mice with reactivation of *Toxoplasma gondii* infection. C: no *T. gondii* + DEX + PBS; G: 3 *T. gondii* cysts + DEX + PBS; S: 3 *T. gondii* cysts + DEX + koumiss; X: 3 *T. gondii* cysts + DEX + mare’s milk. All data are expressed as mean ± SD.

Twenty-eight days after the mice were infected with *T. gondii*, DEX was added to the drinking water of the mice to establish a reactivation of *T. gondii* infection model, and the survival rate of the mice was observed. As shown in Figure 1. All the mice in Group C survived, and the mice in Group G began to die from 6 days after addition of DEX. The mice in Groups S and X died at 8 dpi. At 14 dpi, the survival rates of mice in Groups G, S, and X were 11.1, 22.2, and 11.1%, respectively. There was no significant difference in the survival rates between the different groups.

### The effect of koumiss treatment on SHIRPA in mice

**FIGURE 2:**
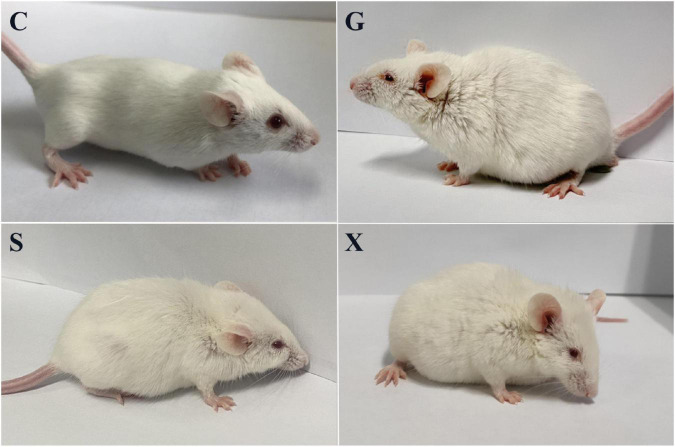
Pictures of mice with reactivation of *Toxoplasma gondii* infection. C: no *T. gondii* + DEX + PBS; G: 3 *T. gondii* cysts + DEX + PBS; S: 3 *T. gondii* cysts + DEX + koumiss; X: 3 *T. gondii* cysts + DEX + mare’s milk.

**FIGURE 3:**
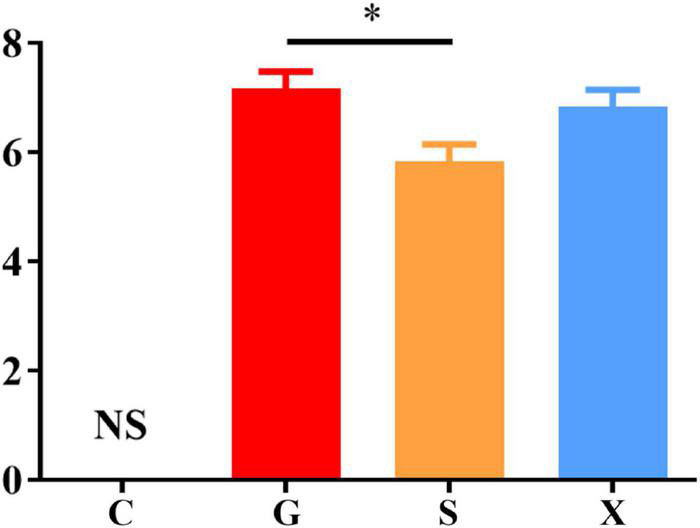
SHIRPA of mice with reactivation of *Toxoplasma gondii* infection. C: no *T. gondii* + DEX + PBS; G: 3 *T. gondii* cysts + DEX + PBS; S: 3 *T. gondii* cysts + DEX + koumiss; X: 3 *T. gondii* cysts + DEX + mare’s milk. All data are expressed as mean ± SD. *P < 0.05 and NS, no significance.

Following treatment with DEX, the function of the immune system of the mice is weakened, and immunity is reduced. The bradyzoites in the mice are activated into tachyzoites, which invade host cells again and cause serious harm, and the originally stable latent infection of *T. gondii* is reactivated to acute infection. Mice develope a series of clinical symptoms, such as pilosis, squinting, tearing, weight loss, diarrhea, slower movement speed, involuntary trembling, and curling up. As shown in Figure 2. The mice in the Group G showed obvious pilosis and curling up, accompanied by severe squinting and tearing. Clinical symptoms resolved in Group X, but slight pilosis, squinting and curling up remained. Compared with Group G, The symptoms of pilosis and curling up in Group S have significantly disappeared. Using the SHIRPA to describe the behavior and symptoms of the mice, different treatment groups had different effects on the SHIRPA (Figure 3). Compared with Group G, the scores of Group S decreased significantly (*P* < 0.05), while compared with Group X, there was no significant difference in scores. From this result we conclude that yogurt treatment has a certain inhibitory effect on the reactivation of *T. gondii* infection in mice.

### Effects of different treatments on serum cytokine levels in mice

**FIGURE 4:**
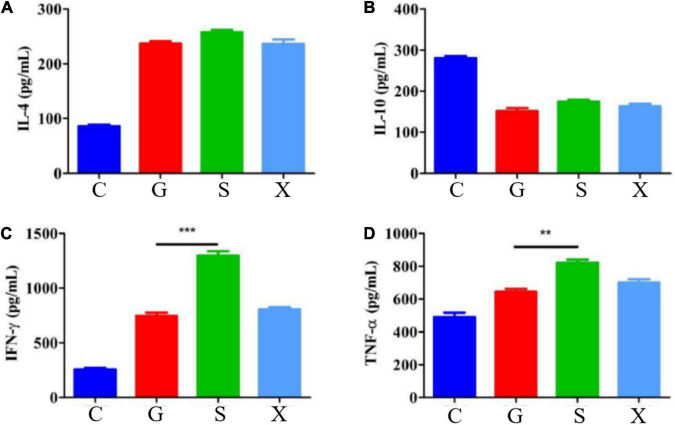
Serum cytokines levels of IL-4, IL-10, IFN-γ, and TNF-α in mice with reactivation of *Toxoplasma gondii*. C: no *T. gondii* + DEX + PBS; G: 3 *T. gondii* cysts + DEX + PBS; S: 3 *T. gondii* cysts + DEX + koumiss; X: 3 *T. gondii* cysts + DEX + mare’s milk; **(A–D)** Indicate IL-4, IL-10, IFN-γ and TNF-α of serum cytokines levels, respectively. All data are expressed as mean ± SD. **P < 0.01; ***P < 0.001.

Mice were treated with DEX to establish a reactivation model of *T. gondii*. Blood was collected from the eyes of the mice at 7 dpi, the blood samples were separated into serum, and the concentrations of the cytokines IL-4, IL-10, IFN-γ, and TNF-α were measured by commercial ELISA kits. Compared with Group C, the concentration of IL-4 increased after mice were infected with *T. gondii*, among which Group S had the highest level, but there was no significant difference between Groups G, S, and X (Figure 4A). After mice were infected with *T. gondii*, the concentration of IL-10 generally showed a decreasing trend, and there was no significant difference among the G, S, and X groups (Figure 4B). Compared with Group C, the levels of IFN-γ in Groups G, S, and X all increased. In terms of the effects of the different treatment methods on IFN-γ, the level of Group S increased the most and was significantly different from that of Group G (*P* < 0.001) but was not significantly different from that of the X group (Figure 4C). Compared with Group C, the levels of TNF-α in Groups G, S and X were all increased, and there was a significant difference between Group S and Group G (*P* < 0.01) but no significant difference from Group X (Figure 4D).

### Koumiss treatment reduces organ parasite burden in mice with reactivation of *Toxoplasma gondii* infection

**FIGURE 5:**
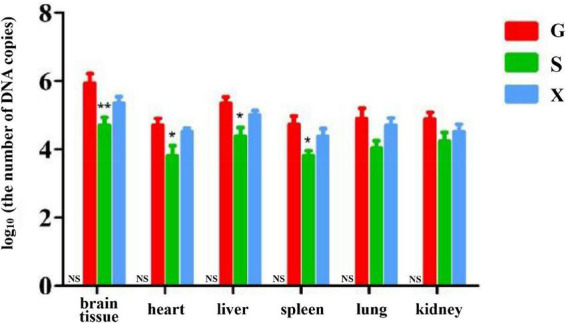
Organ parasite burden of mice with reactivation of *Toxoplasma gondii* infection. C: no *T. gondii* + DEX + PBS; G: 3 *T. gondii* cysts + DEX + PBS; S: 3 *T. gondii* cysts + DEX + koumiss; X: 3 *T. gondii* cysts + DEX + mare’s milk. All data are expressed as mean ± SD. *P < 0.05; **P < 0.01 and NS, no significance between Group C and other groups.

Long-term chronic stable infection developed in mice 28 days after infection with *T. gondii*. Mice were induced with DEX to establish the parasitic protozoa infection reactivation model. The mice in each group were treated as described above. The mice were sacrificed and dissected at 7 dpi. The brain tissue, heart, liver, spleen, lung and kidney of the mice were collected, and genomic DNA was extracted using a tissue genomic DNA extraction kit. RT-PCR was used to determine the relative expression of the *T. gondii* gene in different organs of mice and to determine the parasite burden in different organs. As shown in Figure 5. The DNA copies of *T. gondii* in the brain tissue in Group S were lower than those in Group G, and there was a significant difference between Groups S and G (*P* < 0.01). The DNA copies of *T. gondii* in the heart, liver and spleen of mice in Group S were significantly lower than those in Group G (*P* < 0.05). Compared with Group G, although the number of DNA copies of *T. gondii* in the lungs and kidneys of mice in Group S decreased, there was no significant difference between the different groups. The DNA copies of *T. gondii* in each tissue and organ of mice in Group X were lower than those in Group G, but there was no significant difference between the different treatment groups.

### Effect of koumiss treatment on intestinal microbiota with reactivation of *Toxoplasma gondii* infection

**FIGURE 6:**
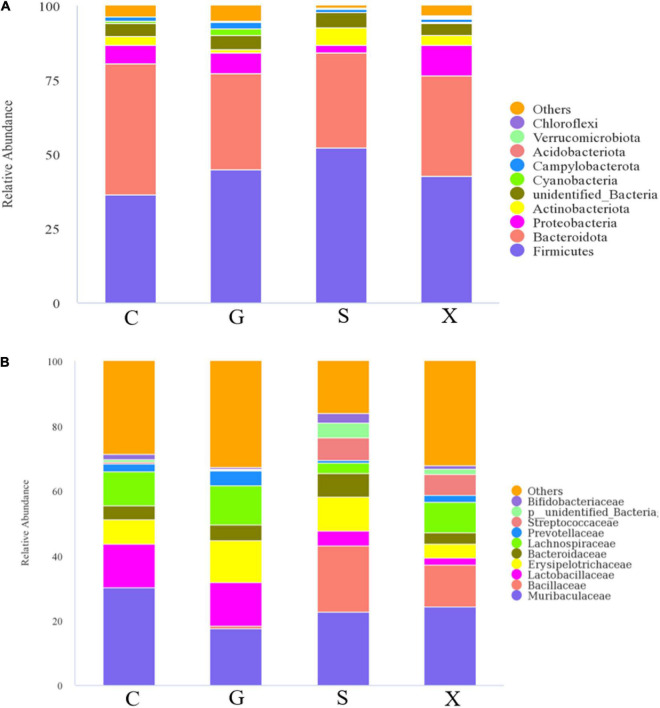
The intestinal microbiota composition changes of mice with reactivation of *Toxoplasma gondii* at 7 dpi. C: no *T. gondii* + DEX + PBS; G: 3 *T. gondii* cysts + DEX + PBS; S: 3 *T. gondii* cysts + DEX + koumiss; X: 3 *T. gondii* cysts + DEX + mare’s milk. **(A,B)** Indicate phylum and family levels, respectively. All data are expressed as mean ± SD.

After the mice were infected with *T. gondii* to form a long-term and stable inapparent infection, the mice were treated with DEX to establish a reactivated *T. gondii* infection model. Feces of mice in each group were collected aseptically after 7 days, and 16S rRNA gene sequencing was performed on their intestinal microbiota. The results are shown in Figure 6. The species composition of the C group consisted of *Bacteroidota* (44.3%), which was the highest, followed by *Firmicutes* (36.3%), *Proteobacteria* (6.2%) and others at the phylum level (Figure 6A). Compared with Group C, the relative abundance of Bacteroidetes in Groups G, S, and X showed a downward trend of 32.4, 32.1, and 33.9%, respectively. The relative abundance of *Firmicutes* increased, of which Group S (52.2%) increased the most. The relative abundance of *Proteobacteria* in Groups G and X increased by 6.9 and 10.0%, respectively, while the relative abundance of *Proteobacteria* in Group S decreased by 2.4%. Compared with Group C, the relative abundance of *Actinobacteriota* in Group G decreased (1.1%), while in Group S, it increased (5.9%). The species composition of the C group consisted of *Muribaculaceae* (30.1%), *Lactobacillaceae* (13.4%), *Lachnospiraceae* (10.3%), and others at the family level (Figure 6B). *Muribaculaceae* was the dominant family in Groups C, G, S, and X, but compared with Group C, the proportion of *Muribaculaceae* in Groups G, S, and X showed a downward trend, and the decrease in Group S was the least. Importantly, compared with Group C, the number of *Bacillaceae* in Groups S and X was increased.

**FIGURE 7:**
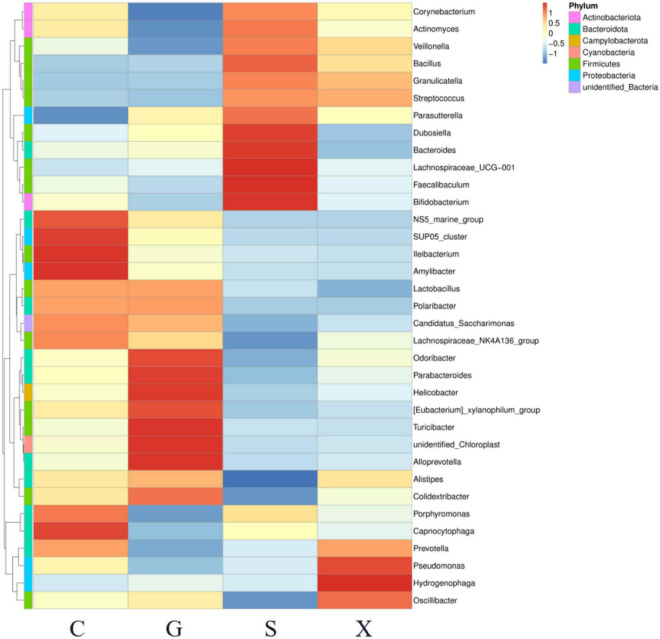
The relationship between the relative abundance of the top 35 genera in the gut microbiota of mice with reactivation of *Toxoplasma gondii* infection at 7 dpi. C: no *T. gondii* + DEX + PBS; G: 3 *T. gondii* cysts + DEX + PBS; S: 3 *T. gondii* cysts + DEX + koumiss; X: 3 *T. gondii* cysts + DEX + mare’s milk. All data are expressed as mean ± SD.

A heatmap showed the relationship between the relative abundance of the top 35 genera in the gut microbiota and different treatment groups (Figure 7). Compared with Group C, the relative abundance of *Corynebacterium*, *Actinomyces*, *Veillonella*, *Faecalibaculum*, and *Bifidobacterium* in Group G was decreased; however, in Group S these decreases were restored and were even higher than that in Group C.

## Discussion

*T. gondii* is an obligate intracellular parasite that infects nucleated cells of any mammalian or avian species (25). Hosts infected with *T. gondii* develop latent infection and show no clinical signs of toxoplasmosis. When host immunity decreases in the state of *T. gondii* latent infection, latent infection is activated, causing severe acute toxoplasmosis again. In clinical studies, patients with solid malignant tumors, solid organ transplant (SOT) (26), human immunodeficiency virus (HIV)/AIDS, diabetes, and those receiving immunosuppressive agents have a high risk of reactivated toxoplasmosis (27, 28). Therefore, reactivation of *T. gondii* infection is more common in patients with these diseases. In this experiment, we used *T. gondii* cysts to infect BALB/c mice. After the formation of latent infection, DEX was used to induce mice to establish a model of *T. gondii* latent infection activation.

In this experiment, the survival rate results showed that there was no significant difference in the survival rate of mice in Groups G, S, and X. However, the results indicated that the treatment of koumiss improved clinical signs of mice. In the SHIRPA test, different treatment groups had different effects on the SHIRPA, and the scores of mice fed koumiss were lower than those of the other treatment groups. Previous studies have shown that sulfadiazine, a traditional drug for the treatment of *T. gondii*, might promote the recovery of microcirculatory homeostasis after infection with *T. gondii* by preventing the neuroinflammatory effects of such focal reinfection (29), and koumiss may also alleviate the clinical symptoms of mice by a similar mechanism. The results of the organ parasite burden of the reactivation of *T. gondii* infection in mice showed that the parasite burden in the brain tissue, heart, liver and spleen of mice was significantly lower than that in the infection group after consuming koumiss. Compared with the parasite burden in other organs, that in brain tissue was always higher. This may brain tissue is the predisposing organ of *T. gondii* (30). This result indicated that although koumiss could not improve the survival rate of infected mice, it could reduce the organ parasite burden and relieve the clinical symptoms, suggesting that koumiss can inhibit the reactivation of *T. gondii* infection to a certain extent.

Serum cytokine levels were determined by ELISA. TNF-α and IFN-γ secreted by Th1 cells are representatives of immune upregulating factors, which have specific chemotactic activation effects on monocytes and macrophages (31). Natural Killer cells (NK cells) have critical protective roles in innate immunity during the *T. gondii* infection through releasing IFN-γ (32). IL-10 secreted by Th2 cells is a representative of immune downregulation factors, which can promote the immune evasion and survival of pathogens such as *T. gondii* (33). The levels of proinflammatory factors and anti-inflammatory factors maintain a relatively stable dynamic equilibrium, and a severe pathological immune responses occurs when this equilibrium is broken (34). IL-10 can inhibit the excessive production of IFN-γ, which causes a severe inflammatory response and even tissue necrosis. IL-10 plays an important role in the host’s resistance to *T. gondii* infection (35). In this study, after *T. gondii* latent infection was reactivated, the level of IFN-γ was significantly increased, which may be due to the breakdown of the dynamic equilibrium between proinflammatory factors and anti-inflammatory factors, the reduction in the concentration of IL-10, and the dysfunction of immune function. Overproduction of IFN-γ leads to relapse of latent infection of *T. gondii*. The concentration of IL-10 increased after feeding koumiss, which may be due to the improvement of the imbalanced immune function and the relief of the reactivation of *T. gondii* infection. This is consistent with the conclusion that koumiss may alleviate the clinical symptoms of reactivation of *T. gondii* infection.

Alterations in host intestinal microbiota were thought to affect host behavior and cognition (36). Previous studies have shown that *T. gondii* infection altered the composition of the host intestinal flora (37). Here, we examined the intestinal microbiota of the reactivation of *T. gondii* infection. Compared with the intestinal flora composition of uninfected control mice, the mice infected with *T. gondii* had more conditional pathogenic bacteria. The results showed that the proportion of *Proteobacteria* in the intestinal flora of infected mice increased slightly. An increasing proportion of *Proteobacteria* will change the overall structure of the intestinal flora. The relative abundance of *Proteobacteria* in many host intestines is thought to be related to the ecological imbalance of the host gut microbiota and can be used as a marker of intestinal flora instability. Previous studies have shown that the severity of colitis is positively correlated with the abundance of *Proteobacteria* (38). The severity of clinical symptoms of recessively infected activated mice treated with koumiss was alleviated, which may be due to the reduction in the proportion of *Proteobacteria* in the intestinal tract, thus reducing body inflammation. The results showed that while the relative abundance of *Proteobacteria* in Group S decreased, the proportion of *Firmicutes* increased, which corresponded to the increase in the relative abundance of *Bacillus* in Group S at the family level. In addition, the relative abundance of *Bifidobacterium* in Group S was increased. Members of the genus *Bifidobacterium* are high G + C Gram-positive bacteria belonging to the phylum *Actinobacteria* and represent common inhabitants of the gastrointestinal tract (GIT) of mammals, birds and certain cold-blooded animals (39). Among the known health-promoting or probiotic microorganisms, bifidobacteria represent one of the most dominant groups, and some bifidobacterial species are frequently used as probiotic ingredients in many functional foods (39). *Bifidobacterium* spp. seems to exert immunomodulatory effects, impacting the immune system both locally and systemically (40). Previous studies have shown that *Bifidobacterium* interacts with human immune cells and modulates specific pathways involved in innate and adaptive immune processes (41). A synbiotic composed of *Bifidobacterium* animalis and fructooligosaccharides is capable of synthesizing IFN-γ and may be beneficial to the control of toxoplasmosis (42). Therefore, we propose that consuming koumiss may improve the composition of intestinal flora structure. *Proteobacteria* was replaced by *Firmicutes*, and the relative abundance of *Bifidobacterium* increased, which inhibited the colonization of *T. gondii* in the host, thus reducing the amount of organ parasite burden and alleviating the severity of clinical symptoms in mice with reactivation of *T. gondii* infection.

Our results showed that koumiss treatment may improve the clinical symptoms of mice and the composition of intestinal flora structure, significantly reducing the organ parasite burden of mice, inhibiting the *T. gondii* latent activation infection, and relieving the symptoms of reactivation of protozoan infection. In this study, we investigated the relationship between intracellular parasitic protozoa and hosts and provided methodologies and evidence for the mitigation and treatment of *T. gondii* infection, as well as broadening the ideas for the application of koumiss and probiotics in the fight against intracellular infection.

## Data availability statement

The data presented in this study are deposited in the NCBI repository (https://www.ncbi.nlm.nih.gov/), accession number: PRJNA873296.

## Ethics statement

This animal study was reviewed and approved by the Inner Mongolia Agricultural University Laboratory Animal Welfare and Animal Experimental Ethical Inspection Committee (NND2021069).

## Author contributions

XYa and YS: study concept and design. YS, XYu, JG, RL, XJ, WH, WG, PL, and JC: samples collection and experiment. XYa, YS, and HW: analysis and interpretation of data. YS and XYu: manuscript writing. XYa: manuscript review and editing, supervision, funding acquisition, and project administration. All authors contributed to the article and approved the submitted version.

## Footnotes

1
http://ccb.jhu.edu/software/FLASH/

2
http://qiime.org/scripts/

3
https://www.arb-silva.de/

4
http://www.drive5.com/uparse/

## References

[1] Liu Q Wang ZD Huang SY Zhu XQ. Diagnosis of toxoplasmosis and typing of *Toxoplasma gondii*. *Parasite Vector.* (2015) 8:1–14. 10.1186/s13071-015-0902-6 26017718 PMC4451882

[2] Saadatnia G Golkar M. A review on human toxoplasmosis. *Scand J Infect Dis.* (2012) 44:805–14. 10.3109/00365548.2012.693197 22831461

[3] Dubey JP. History of the discovery of the life cycle of *Toxoplasma gondii*. *Int J Parasitol.* (2009) 39:877–82. 10.1016/j.ijpara.2009.01.005 19630138

[4] Werk R. How does *Toxoplasma gondii* enter host cells? *Rev Infect Dis.* (1985) 7:449–57. 10.1093/clinids/7.4.449 3898305

[5] Zhou P Chen N Zhang RL Lin RQ Zhu XQ. Food-borne parasitic zoonoses in China: perspective for control. *Trends Parasitol.* (2008) 24:190–6. 10.1016/j.pt.2008.01.001 18314393

[6] Ayi I Kwofie KD Blay EA Osei JHN Frempong KK Koku R Clonal types of Toxoplasma gondii among immune compromised and immune competent individuals in Accra, Ghana. *Parasitol Int.* (2016) 65:238–44. 10.1016/j.parint.2016.01.004 26775819

[7] Pereira-Chioccola VL Vidal JE Su C. *Toxoplasma gondii* infection and cerebral toxoplasmosis in HIV-infected patients. *Fut Microbiol.* (2009) 4:1363–79. 10.2217/fmb.09.89 19995194

[8] Horacek J Flegr J Tintera J Verebova K Spaniel F Novak T Latent toxoplasmosis reduces gray matter density in schizophrenia but not in controls: voxel-based-morphometry (VBM) study. *World J Biol Psychiatry.* (2012) 13:501–9. 10.3109/15622975.2011.573809 21599563

[9] Yolken RH Dickerson FB Fuller Torrey E. Toxoplasma and schizophrenia. *Parasite Immunol.* (2009) 31:706–15. 10.1111/j.1365-3024.2009.01131.x 19825110

[10] Kodym P Malý M Beran O Jilich D Rozsypal H Machala L Incidence, immunological and clinical characteristics of reactivation of latent *Toxoplasma gondii* infection in HIV-infected patients. *Epidemiol Infect.* (2015) 143:600–7. 10.1017/S0950268814001253 24850323 PMC9507066

[11] Cani PD. Gut cell metabolism shapes the microbiome. *Science.* (2017) 357:548–9. 10.1126/science.aao2202 28798116

[12] Gubert C Kong G Renoir T Hannan AJ. Exercise, diet and stress as modulators of gut microbiota: Implications for neurodegenerative diseases. *Neurobiol Dis.* (2020) 134:104621. 10.1016/j.nbd.2019.104621 31628992

[13] Bercik P Verdu EF Foster JA Macri J Potter M Huang X Chronic gastrointestinal inflammation induces anxiety-like behavior and alters central nervous system biochemistry in mice. *Gastroenterology.* (2010) 139:2102–12. 10.1053/j.gastro.2010.06.063 20600016

[14] Afzaal M Saeed F Anjum F Waris N Husaain M Ikram A Nutritional and ethnomedicinal scenario of koumiss: A concurrent review. *Food Sci Nutr.* (2021) 9:6421–8. 10.1002/fsn3.2595 34760271 PMC8565204

[15] Guo L Ya M Guo YS Xu WL Li CD Sun JP Study of bacterial and fungal community structures in traditional koumiss from Inner Mongolia. *J Dairy Sci.* (2019) 102:1972–84. 10.3168/jds.2018-15155 30639001

[16] Li C Liu X Wang H Fan H Mi Z Kwok LY Koumiss consumption induced changes in the fecal metabolomes of chronic atrophic gastritis patients. *J Funct Foods.* (2019) 62:103522. 10.1016/j.jff.2019.103522

[17] Koppe L Mafra D Fouque D. Probiotics and chronic kidney disease. *Kidney Int.* (2015) 88:958–66. 10.1038/ki.2015.255 26376131

[18] Rong J Zheng H Liu M Hu X Wang T Zhang X Probiotic and anti-inflammatory attributes of an isolate *Lactobacillus helveticus* NS8 from Mongolian fermented koumiss. *BMC Microbiol.* (2015) 15:196. 10.1186/s12866-015-0525-2 26428623 PMC4591576

[19] Gesudu Q Zheng Y Xi X Hou QC Xu H Huang W Investigating bacterial population structure and dynamics in traditional koumiss from Inner Mongolia using single molecule real-time sequencing. *J Dairy Sci.* (2016) 99:7852–63. 10.1186/s12866-015-0525-2 27522429

[20] Bao WYDL He YX Liu W. Diversity analysis of bacterial and function prediction in hurunge from mongolia. *Front Nutr.* (2022) 9:835123. 10.3389/fnut.2022.835123 35399660 PMC8990233

[21] Zhang J Qin X Zhu YU Zhang S Zhang XW Lu HE. Mechanism of dexamethasone in the context of *Toxoplasma gondii* infection. *Parasitology.* (2017) 144:1551–9. 10.1017/S0031182017001111 28653591

[22] Rogers DC Fisher EMC Brown SDM Peters J Hunter AJ Martin JE. Behavioral and functional analysis of mouse phenotype: SHIRPA, a proposed protocol for comprehensive phenotype assessment. *Mamm Genome.* (1997) 8:711–3. 10.1007/s003359900551 9321461

[23] Li X Chen S Huang S Lu F. Mast cell activator compound 48/40 is not an effective adjuvant for UV-attenuated *Toxoplasma gondii* vaccine. *Parasitol Res.* (2017) 116:2347–53. 10.1007/s00436-017-5522-y 28573462

[24] Li W Xu XL Zhang J Cai JH Tang YX. Effects of cyclooxygenase inhibitors on survival time in ovarian cancer xenograft-bearing mice. *Oncol Lett.* (2012) 4:1269–73. 10.3892/ol.2012.929 23205124 PMC3506753

[25] Calero-Bernal R Gennari SM. Clinical toxoplasmosis in dogs and cats: an update. *Front Vet Sci.* (2019) 6:54. 10.3389/fvets.2019.00054 30863754 PMC6399377

[26] Tan S Tong WH Vyas A. Impact of plant-based foods and nutraceuticals on *Toxoplasma gondii* cysts: nutritional therapy as a viable approach for managing chronic brain toxoplasmosis. *Front Nutr.* (2022) 9:827286. 10.3389/fnut.2022.827286 35284438 PMC8914227

[27] Wang ZD Wang SC Liu HH Ma HY Li ZY Wei F Prevalence and burden of *Toxoplasma gondii* infection in HIV-infected people: a systematic review and meta-analysis. *Lancet HIV.* (2017) 4:e177–88. 10.1016/S2352-3018(17)30005-X 28159548

[28] Khattab HM El Bassiouni SO Abuelela MH Abd Elsalam DO. Seroprevalence of *Toxoplasma gondii* among a group of Egyptian patients with type I diabetes mellitus. *Bull Natl Res Centre.* (2019) 43:1–7. 10.1186/s42269-019-0059-0

[29] Estato V Stipursky J Gomes F Mergener TC Frazão-Teixeira E Allodi S The neurotropic parasite *Toxoplasma gondii* induces sustained neuroinflammation with microvascular dysfunction in infected mice. *Am J Pathol.* (2018) 188:2674–87. 10.1016/j.ajpath.2018.07.007 30121257

[30] Juránková J Basso W Neumayerová H Baláž V Jánová E Sidler X Brain is the predilection site of *Toxoplasma gondii* in experimentally inoculated pigs as revealed by magnetic capture and real-time PCR. *Food Microbiol.* (2014) 38:167–70. 10.1016/j.fm.2013.08.011 24290640

[31] de Lemos JA Morrow DA Sabatine MS Murphy SA Gibson CM Antman EM Association between plasma levels of monocyte chemoattractant protein-1 and long-term clinical outcomes in patients with acute coronary syndromes. *Circulation.* (2003) 107:690–5. 10.1161/01.cir.0000049742.68848.99 12578870

[32] Mahmoudzadeh S Nozad CH Marques CS Bahadory S Ahmadpour E. The role of IL-12 in stimulating NK cells against *Toxoplasma gondii* infection: a mini-review. *Parasitol Res.* (2021) 120:2303–9. 10.1007/s00436-021-07204-w 34110502

[33] Jeong YI Hong SH Cho SH Park MY Lee SE. Induction of IL-10-producing regulatory B cells following *Toxoplasma gondii* infection is important to the cyst formation. *Biochem Biophys Rep.* (2016) 7:91–7. 10.1016/j.bbrep.2016.05.008 28955894 PMC5613251

[34] Hedegaard CJ Krakauer M Bendtzen K Lund H Sellebjerg F Nielsen CH. T helper cell type 1 (Th1), Th2 and Th17 responses to myelin basic protein and disease activity in multiple sclerosis. *Immunology.* (2008) 125:161–9. 10.1111/j.1365-2567.2008.02837.x 18397264 PMC2561132

[35] Iyer SS Cheng G. Role of interleukin 10 transcriptional regulation in inflammation and autoimmune disease. *Crit Rev Immunol.* (2012) 32:23–63. 10.1615/CritRevImmunol.v32.i1.30 22428854 PMC3410706

[36] Prandovszky E Li Y Sabunciyan S Steinfeldt CB Avalos LN Gressitt KL *Toxoplasma gondii*-induced long-term changes in the upper intestinal microflora during the chronic stage of infection. *Scientifica.* (2018) 2018:2308619. 10.1155/2018/2308619 30515345 PMC6236704

[37] Shao DY Bai X Tong MW Zhang YY Liu XL Zhou YH Changes to the gut microbiota in mice induced by infection with *Toxoplasma gondii*. *Acta Trop.* (2020) 203:105301. 10.1016/j.actatropica.2019.105301 31843385

[38] Alkadhi S Kunde D Cheluvappa R Randall-Demllo S Eri R. The murine appendiceal microbiome is altered in spontaneous colitis and its pathological progression. *Gut Pathog.* (2014) 6:1–10. 10.1186/1757-4749-6-25 25002910 PMC4085080

[39] Turroni F Van Sinderen D Ventura M. Genomics and ecological overview of the genus *Bifidobacterium*. *Int J Food Microbiol.* (2011) 149:37–44. 10.1016/j.ijfoodmicro.2010.12.010 21276626

[40] Kaźmierczak-Siedlecka K Roviello G Catalano M Polom K. Gut microbiota modulation in the context of immune-related aspects of *Lactobacillus* spp. and *Bifidobacterium* spp. in Gastrointestinal Cancers. *Nutrients.* (2021) 13:2674. 10.3390/nu13082674 34444834 PMC8401094

[41] Ruiz L Delgado S Ruas-Madiedo P Sánchez B Margolles A. *Bifidobacteria* and their molecular communication with the immune system. *Front Microbiol.* (2017) 8:2345. 10.3389/fmicb.2017.02345 29255450 PMC5722804

[42] Ribeiro CM Costa VM Gomes MIFV Golim MA Modolo JR Langoni H. Effects of synbiotic-based *Bifidobacterium animalis* in female rats experimentally infected with *Toxoplasma gondii*. *Comp Immunol Microbiol.* (2011) 34:111–4. 10.1016/j.cimid.2010.03.002 20409588

